# A Framework for Quantitative Modeling of Neural Circuits Involved in Sleep-to-Wake Transition

**DOI:** 10.3389/fneur.2015.00032

**Published:** 2015-02-26

**Authors:** Siamak Sorooshyari, Ramón Huerta, Luis de Lecea

**Affiliations:** ^1^Bell Laboratories, Alcatel-Lucent, Murray Hill, NJ, USA; ^2^BioCircuits Institute, University of California San Diego, La Jolla, CA, USA; ^3^Department of Psychiatry and Behavioral Sciences, Stanford University School of Medicine, Stanford, CA, USA

**Keywords:** hypocretin, orexin, hypothalamus, neuromodulation, awakenings, optogenetics, arousal system

## Abstract

Identifying the neuronal circuits and dynamics of sleep-to-wake transition is essential to understanding brain regulation of behavioral states, including sleep–wake cycles, arousal, and hyperarousal. Recent work by different laboratories has used optogenetics to determine the role of individual neuromodulators in state transitions. The optogenetically driven data do not yet provide a multi-dimensional schematic of the mechanisms underlying changes in vigilance states. This work presents a modeling framework to interpret, assist, and drive research on the sleep-regulatory network. We identify feedback, redundancy, and gating hierarchy as three fundamental aspects of this model. The presented model is expected to expand as additional data on the contribution of each transmitter to a vigilance state becomes available. Incorporation of conductance-based models of neuronal ensembles into this model and existing models of cortical excitability will provide more comprehensive insight into sleep dynamics as well as sleep and arousal-related disorders.

## Introduction

A primary objective of this paper is to spawn motivation for analytical modeling in the study of sleep-to-wake transitions. In effect, we aim to provide a bridge between conventional empirical studies that have been governed by the biological sciences (including psychiatry) and a system-theoretic approach, which is largely algorithmic. It is possible that subsets of the problems discussed in this work are discovered to not have a unique or tangible solution. While not revolutionary from an engineering perspective and of limited short-term value to an empirical neuroscientist; such analysis is necessary in the scientific process of developing testable hypotheses. Two crucial points must be considered with respect to the scope of this paper. First, the specific system in question is the assembly of neural circuits involved in the sleep-to-wake cycle. We will use the terms system and network interchangeably to refer to anatomical units within a biological domain. Second, the majority of our references will be to findings obtained via an interrogation of neuronal systems by optogenetic experiments as well as single/multi unit recordings (1–5) or acquisition of calcium imaging data. Optogenetics has been acclaimed as a valuable tool for dissecting the interaction of neural circuits in the regulation of sleep and wakefulness, and allows interrogation of genetically defined cell types with millisecond precision (6, 7).

The hypocretin (Hcrt) system is a logical starting point to develop models of sleep/wake cycle (8, 9). Specifically, works such as Peyron et al. (10) and Chemelli et al. (11) have reported malfunction of the Hcrt network being associated with fragmented sleep and wake states symptomatic of narcolepsy and cataplexy in humans, dogs, and mice. Hcrt-producing neurons fire phasically in anticipation and during active wakefulness whereas they are mostly quiescent during quiet waking, NREM, and REM sleep (2, 12). This suggests that the main function of the Hcrt system is to control boundaries between vigilance states. Therefore, we will focus this initial model on sleep-to-wake transitions. In addition to sleep and wakefulness, the Hcrt system has been linked with a myriad of physiological functions (13), largely attributed to the localization of Hcrt neurons in the lateral hypothalamus and their expansive projections throughout the brain. The projections include the locus coeruleus (LC), dorsal raphe nucleus, laterodorsal tegmentum, ventral tegmental area (VTA), tuberomammillary nucleus, and basal forebrain (BF) (9, 14). A deeper understanding of the Hcrt system also has translational significance, as Hcrt receptor antagonist drugs that specifically block Hcrt neurotransmission are undergoing approval for clinical use for primary insomnia (15, 16). Although we will not specifically address the role of individual Hcrt receptors (Hcrtr1 and Hcrtr2) in sleep/wake cycles, this component may be added to the model in the future. Furthermore, numerous pathologies such as chronic stress (17), panic and post-traumatic stress disorders (18, 19), and reinstatement of drug seeking behavior (20–22) may be linked to alterations in Hcrt transmission.

Transitions between sleep states are routinely monitored by electroencephalography (EEG), which records electrical activity as a result of the flow of ionic currents throughout the cortex (23). The NREM sleep state is characterized by high amplitude and low frequency slow waves in the EEG, whereas in the awake state fast activity at high frequencies and low amplitudes emerge. REM sleep is also characterized by fast activity, mostly in the theta and gamma band, and loss of muscle tone (24, 25). The transition between sleep and wakefulness has been shown to involve fast glutamate transmission and wake-promoting neuromodulators including norepinephrine (NE), acetylcholine (ACh), serotonin (5-HT), and histamine (His) (26, 27). It has been recognized that new technology is needed to determine relationships between sleep and wake-promoting neural circuits and the manner in which the lateral hypothalamus, LC, dorsal raphe nucleus, laterodorsal tegmentum, VTA, tuberomammillary nucleus, and BF circuits interact to govern the sleep–wake transition.

We believe that analytical modeling should have a vital role in interpreting, assisting, and driving sleep and neural circuit research. A description of the three terms as follows. First, an analytical model can be used to interpret available data. Presuming the model provides an adequate fit to empirical data; it can be utilized to explain facets of the biological process. An example of this has been the incorporation of point processes to reproduce the spiking activity of neurons based on a neuron’s spiking history, ensemble activity, and extrinsic covariate effects (28). Second, quantitative analysis can assist in the understanding of a biological process through the optimization of fungible model variables along the successive experiments. The free parameters may range from attributes such as the intensity and duration of a stimulus, the intrinsic conductance of the neuron, to the dimensionality of the biological system (i.e., the number of neural circuits) that is considered in the experiment. Within the assist rubric, computational models may also be harnessed to approximate or propose limits on the parameters in a biological system. Lastly, analytical modeling can drive the design of experiments and technologies. For instance, we will discuss how the presented themes of hierarchy, feedback, and redundancy are algorithmic notions, which are suggestive of a future sequence of combinatorial optogenetic experiments.

## Goals and Methodology

To explain the interaction of neural circuits that promote sleep and wakefulness, it is necessary to balance experimental and theoretical approaches. Thus, it becomes important to discuss a distinction between two directions that follow the “*interpret*, *assist*, and *drive*” themes mentioned above. The first involves presenting insightful quantitative analysis which fits available data and explains the interactions between components of the sleep–wake circuitry. This direction pertains mostly to the “*interpret*” theme. The second avenue is the development of analytical models to guide experimenters in designing, adjusting, or validating sleep–wake experiments. Such a direction primarily corresponds to the “*assist*” and “*drive*” notions.

Accordingly, we approach our objective of providing insight into the importance of formulating concrete problems that can be analytically pursued within the sleep-to-wake paradigm. There are two immediate goals that modelers should consider when striving to make computationally oriented contributions to the sleep community.

Goal 1: provide a system-theoretic illustration that concretely depicts the synthesis of the anatomical units based on their respective functions. A subsequent phase is to mathematically represent the input and output signals of the system components.
Goal 2: isolate specific portions of the system, which yield probabilistic responses as a consequence of the stimuli in question. A judicious subsequent phase to this goal would be to determine the probability distributions of the aforementioned stochastic processes via empirical data.

The relatively recent application of optogenetics within the neural circuits involved in the sleep-to-wake cycle (29, 30) has proved illuminating and brought forth new challenges (31). An interesting nuance of modeling the use of optogenetics within this realm is the need to concurrently consider three levels of the nervous system: the neuronal, neural circuit, and behavioral levels. As noted in numerous reviews, the application of optogenetics allows for temporally precise manipulation of electrical and biochemical events while maintaining cell-level resolution. A distinction is made from the neuronal level by considering a neural circuit and the interactions among multiple neural circuits [e.g., one circuit gating another; (32)]. Optogenetics permits a subset of the neural circuits to be targeted while the remaining circuits are left unperturbed. The advent of new imaging technologies that include cell-type recordings using genetically encoded calcium sensors will surely increase the pool and accuracy of data collection relevant to circuit dynamics. Lastly, it is essential to subsume behavior as part of the discussion. This is because the induction and maintenance of sleep are behavioral responses, which can be optogenetically induced (or modulated) via sufficient stimulation/inhibition.

For simplicity, when considering a sleep-to-wake transition we shall restrict attention to a NREM-to-wake transition. It has been acknowledged that transitions among other vigilance states (i.e., NREM-to-REM, wake-to-NREM, REM-to-wake) may involve disparate neurotransmitters, but will likely abide to similar dynamic principles. The neurotransmitters listed in Table 1 are presumed to be the prominent players in this study. In light of this, a brief list of the brain regions containing the neurons which release the neurotransmitters during the sleep-to-wake transition is also provided in Table 1. While pharmacological techniques have been used in studies involving the neural circuit and behavioral levels; the use of optogenetics begets the exciting avenue of concurrent experimentation of the three levels. It is expected that the resultant interaction between the neuronal, neural circuit, and behavioral levels will complicate the prospective research. Our exposition is undertaken with the intent of facilitating future research via a concrete framework to guide experimenters and modelers.

**Table 1:** **A brief account of the neural circuits and neurotransmitters considered in this work**.

Neurotransmitter	Brain region	Notes
His	Tuberomammillary nucleus	Increased during wake periods
		Histamine-deficient mice (HDC ko) show abnormal architecture
		Histamine receptor antagonists increase sleep amounts
		Broad, diffuse projections throughout the brain
		Express mostly Hcrtr2 receptors
NE	Locus coeruleus	Tonic activity (2–3 Hz) during wake periods
		Quiescent during NREM and REM
		Optogenetic activation of LC is sufficient for wakefulness
		Optogenetic inhibition of LC increases amount of sleep
		Strong innervations of neocortex
		Expresses only Hcrtr1 receptors
Hcrt	Lateral hypothalamus	Dysfunction of Hcrt system leads to narcolepsy with cataplexy
		Phasic activity precedes sleep-to-wake transitions
		Quiescent during NREM and REM
		Optogenetic and pharmacogenetic stimulation during sleep increases probability of sleep-to-wake transitions
		Suppresses REM sleep
		Stabilizes wake states by projecting to other neural populations associated with arousal
5-HT	Dorsal raphe	Increased during wake periods
	Raphe magnus	Pharmacologic increase in 5-HT (SSRIs) suppresses REM sleep
		Also affects thermoregulation and respiratory function
		Express Hcrtr1 and Hcrtr2 receptors.
ACh	Basal forebrain	Increased firing during wake and REM periods
	Mesencephalic (LDT and PPTN)	Innervate septum, hippocampus, and cortical neurons
		Express Hcrtr1 and Hcrtr2 receptors
		Fire at low frequencies during NREM
		Increased during the transition to REM sleep and waking
DA	Ventral tegmental area	Increased during wake periods and REM sleep
		Decreased during NREM sleep
		DATko mice show increased sleep amounts
		Express Hcrtr1 and Hcrtr2 receptors
	Substantia nigra	SN projects to striatum. Involved in motor control and habit formation
		Lesions of SN or GP attenuate wakefulness

## Formulation of Computational Models: Hierarchy in Gating, Feedback, and Redundancy

It is imperative to develop an iterative dialog between theoretical models and experimental work. We advocate models that illustrate interactions between various circuits involved in sleep-to-wake transitions. The notions presented in this section are an endeavor to quantitatively represent the empirically determined interactions between several neural circuits. This section constitutes a step toward addressing Goal 1 and Goal 2 of the previous section. In the ensuing subsections, the notions of hierarchy, feedback, and redundancy will be discussed from a network-theoretic perspective.

### A note on several extant models

Although an overview of existing works is not the aim of this paper; it is worthwhile to comment on several extant models that have motivated the present formulation. The seminal work of Saper and colleagues (33–35) presented the flip–flop model of sleep state transition by considering two populations of mutually inhibiting neurons: sleep-promoting and arousal-promoting neurons. In effect, the flip–flop model mirrors a switch since there are two discrete states with the circuitry representing the two neuronal populations. A similar flip/flop mechanism has also been proposed for the regulation of REM sleep, consisting of mutually inhibitory REM-off and REM-on areas in the mesopontine tegmentum (35, 36). The model has been more recently extended to incorporate the homeostatic, circadian, and allostatic factors. The flip–flop model does not readily incorporate the concept of redundancy as discussed in Ref. (37), or the notion of each neurotransmitter contributing in different ways to the dynamics of the transition (e.g., integration, coincidence detection, priming). A consequence of the different effects of the computations performed by the various neurotransmitters will give rise to the notion of hierarchy. We advocate the utility and insight provided by the flip–flop model and do not proclaim the framework introduced here as a substitute, but rather as a more general approach for representing the interactions among the neural circuits and the resultant impact on the behavioral state. Similarly, the limit cycle reciprocal interaction model (LCRIM) has considered dynamic system modeling and the consideration of feedback in the circadian modulation of the REM sleep (38, 39). The framework, and corresponding notion of feedback, presented herein is geared more so toward sleep-to-wake transition [rather than sleep homeostasis as conducted by Postnova et al. (40), Fulcher et al. (41)] and will consider the Hcrt system as well as the incorporation of optogenetics. The primary focus of our effort is to elucidate the complex interactions between the neural circuits involved in the sleep and arousal states. Optogenetics tremendously abets such an attempt by allowing populations of neurons to be selectively stimulated/inhibited so the resultant influence on other circuits and the sleep-to-wake cycle can be studied (31).

### Modeling neural circuit interactions during sleep-to-wake transition

**Figure 1:**
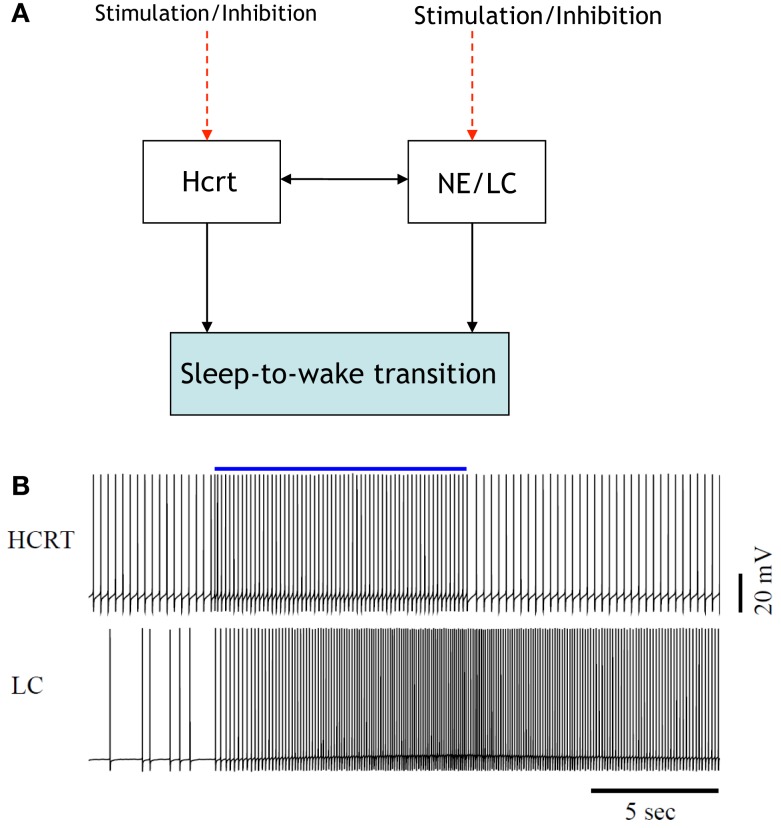
[**(A)**, Top] illustration of the interactions among two neural circuits involved in sleep-to-wake transition. The red dashed lines denote the application of optogenetic signals for the purpose of inducing a sleep-to-wake transition (30, 32). [**(B)**, Bottom] voltage traces of a conductance-based model of an Hcrt-NE/LC circuit comprised 10 Hcrt and 10 NE/LC neurons. One random neuron from the Hcrt and NE/LC populations were chosen for this figure. The Hcrt neurons are stimulated for 10 s (the blue line denotes the occurrence and duration of the stimulation) using optogenetic stimulation as described in Ref. (32). Mechanism for hypocretin-mediated sleep-to-wake transitions, due to the slow activation of the hypocretin receptor on the NE/LC neurons, the firing frequency of the NE/LC neurons increase with a delay and stay active for more than a minute. The hypocretin receptor has the effect of a delayed and prolonged depolarization on the NE/LC neurons.

Analytical representation of previously unmodeled phenomena requires a level of abstraction as a starting point. In engineering, the abstraction is frequently accomplished by providing a block diagram exhibiting the interaction of components within the network. Paramount to such representation is an account of the coupling between the various components. In the sleep–wake system, the coupling is inherent since it has been formed by nature rather than optimized through an engineering procedure. This principle tremendously complicates an in-depth analysis of the system. Figure 1 is a simple illustration of interactions among several neuronal circuits in a sleep-to-wake transition. The shaded box in the figure denotes a behavioral event while the unshaded boxes depict neural circuits. The arrows represent neuronal projections between the circuits and include both fast neurotransmission (i.e., via glutamate) and neuromodulation (via neurotransmitters, modulators, peptides). We suggest the expansion of such a figure and its constituent components into a more detailed architecture as an avenue for future research. For instance, serotonin should take part in the interactions as well, and some efforts have been made to model the interactions between 5-HT-expressing neurons and Hcrt (42). Consensus on more detailed architectures will require coordination between performing experiments and interpreting the findings in a succinct and elucidating manner.

**Figure 2:**
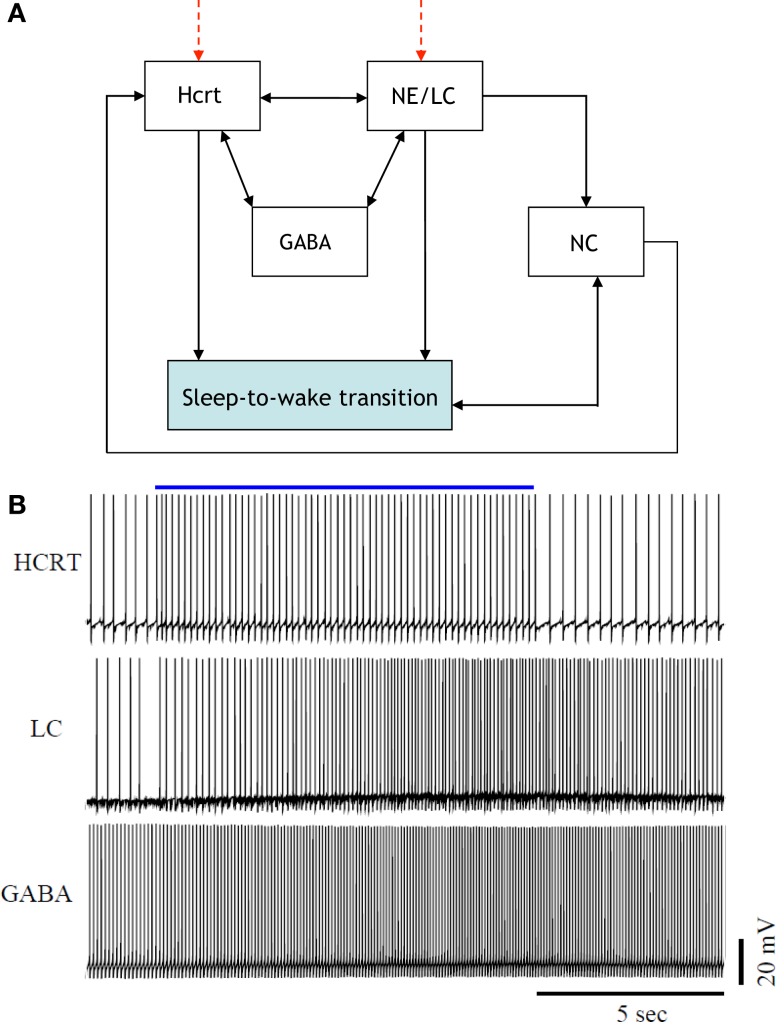
[**(A)**, Top] a more elaborate depiction of the coupling among the neural circuits involved in sleep-to-wake transition including GABA releasing neurons and neurons in the neocortex (NC). Evidence for the above interactions has recently been provided in Ref. (32). [**(B)**, Bottom] implementation of a model of 50 neurons including GABAergic cells. IPSPs can be observed on the individual membrane potential traces of the Hcrt and the NE/LC neurons. GABAergic circuits increase the coherence of oscillation frequencies of Hcrt and NE producing LC neurons.

**Figure 3:**
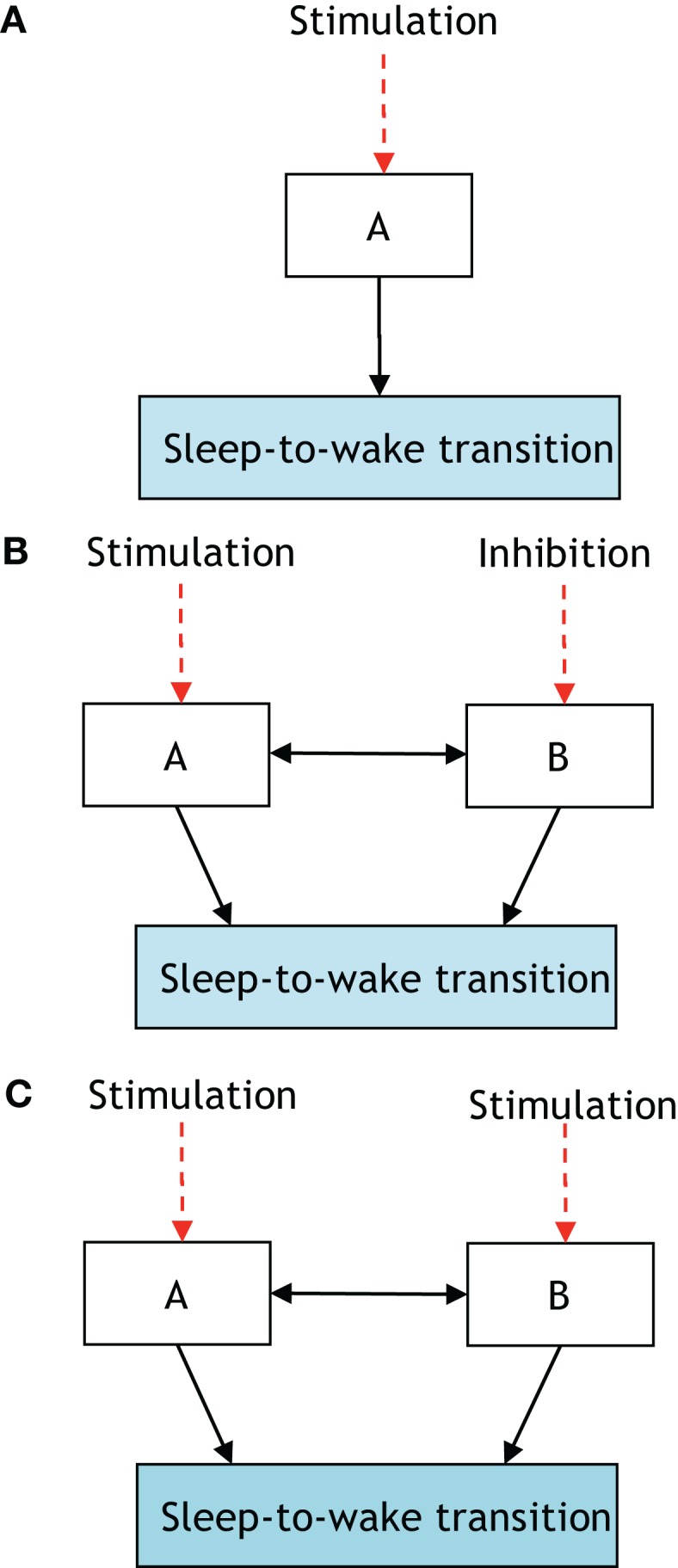
**The presented definition of a neural circuit hierarchically gating another neural circuit in inducing a sleep-to-wake transition**. P[S-to-W] denotes the probability of a sleep-to-wake transition occurring at a given time. **(A)** Consists of one neural circuit being optogenetically stimulated with a resultant P[S-to-W] = *a*. In **(B)**, two neural circuits are considered with population A being stimulated and population B receiving inhibition; the net result is quantified via the probability P[S-to-W] = *b*. Finally, **(C)** shows the same two neural circuits being stimulated and a recording of P[S-to-W] = *c*. If *a* > *b* and *c* > *a* then it will be stated that neural circuit B is hierarchically gating neural circuit A in inducing a sleep-to-wake transition. In Ref. (32), it was empirically determined using optogenetic stimulation/inhibition that the above functional relationship holds with neural circuit A corresponding to Hcrt-releasing neurons, neural circuit B corresponding to NE/LC, and the prospective sleep-to-wake transitions occurring during NREM sleep.

Figure 2 is a more elaborate version of Figure 1 and includes several additional neuronal circuits believed to be involved during sleep-to-wake transitions, namely, GABA-producing neurons and neurons in the neocortex (NC). NE producing neurons in the LC (denoted hereafter as NE/LC) provide extensive innervation to cortical neurons, and NE release is mostly excitatory. Hence, the NC is believed to be a necessary effector of NE neurons (43). Furthermore, the NC is the primary brain region that conveys the oscillations associated with sleep; this has been shown through EEG recordings. GABA circuits are essential for providing control and stability of neuronal networks (44–46). While we acknowledge the diversity and complexity of GABA circuits, we will focus here on populations of local GABA cells that provide feedback control of principal excitatory cells. For instance, MCH neurons and LepRB cells are two essentially different populations of GABA cells that innervate Hcrt cells locally (47). Optogenetic activation of MCH neurons has recently been shown to increase sleep (48, 49). While these two systems may respond to different inputs and affect the circuit dynamic of Hcrt neurons in different ways, their common function as it pertains to Hcrt regulation may be pooled into a single system. These pools may be separated at later stages if there is experimental evidence showing separable dynamic properties (e.g., expression of long-lasting neuromodulators in one population but not in the other population). It should be noted here that conductance-based models using fast GABAA neurons interacting with the Hcrt neurons show that GABAA neurons are not sufficient to control Hcrt/LC activity. GABAA neurons may actually induce stronger Hcrt activity due to synchronization phenomena. Mosqueiro et al. (50) propose a simple alternative mechanism based on the presence of inhibitory neuropeptides that better regulate the system on the same time scales as the Hcrt receptors. While additional regulatory circuits cannot be ruled out, the model itself can control Hcrt and LC output only regulated by GABA neurons. Figure 2 does not illustrate that NE/LC is more potent at eliciting sleep-to-wake transitions than Hcrt (32). It is important to develop a model that provides analytical insight into the fact that NE/LC–Hcrt interaction contains a level of hierarchically with NE/LC gating Hcrt in eliciting sleep-to-wake transitions. In Figure 3, we provide an explicit definition of what it means for a neural circuit to hierarchically gate another neural circuit in the induction of a sleep-to-wake transition. This is a specific concept, which has been empirically investigated through optogenetic experimentation (32). In effect, LC drives the NC activity that is believed to result in the sleep-to-wake transition.

**Figure 4:**
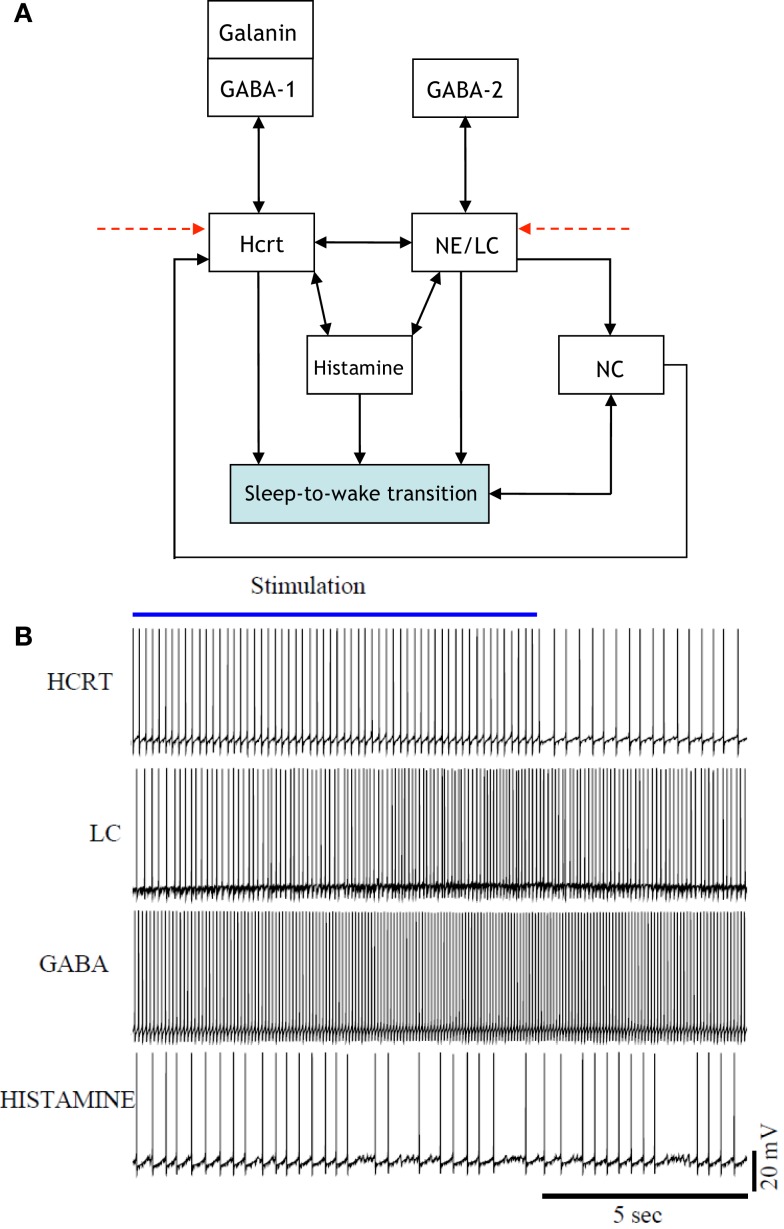
[**(A)**, Top] updated schematic showing the interaction of histamine producing neurons, two populations of GABAergic neurons (GABA-1 and GABA-2), and a galanin producing population in the induction of a sleep-to-wake transition. [**(B)**, Bottom] voltage traces of four neurons chosen at random from the Hcrt, NE/LC, GABAergic, and His populations. The voltage traces were obtained from a realistic model comprised of 60 neurons. The histamine producing neurons were controlled by the excitatory input from the optogenetically stimulated Hcrt-producing neurons and the inhibitory effect of the GABA populations, hence they do not overexcite the NE/LC and Hcrt populations.

**Figure 5:**
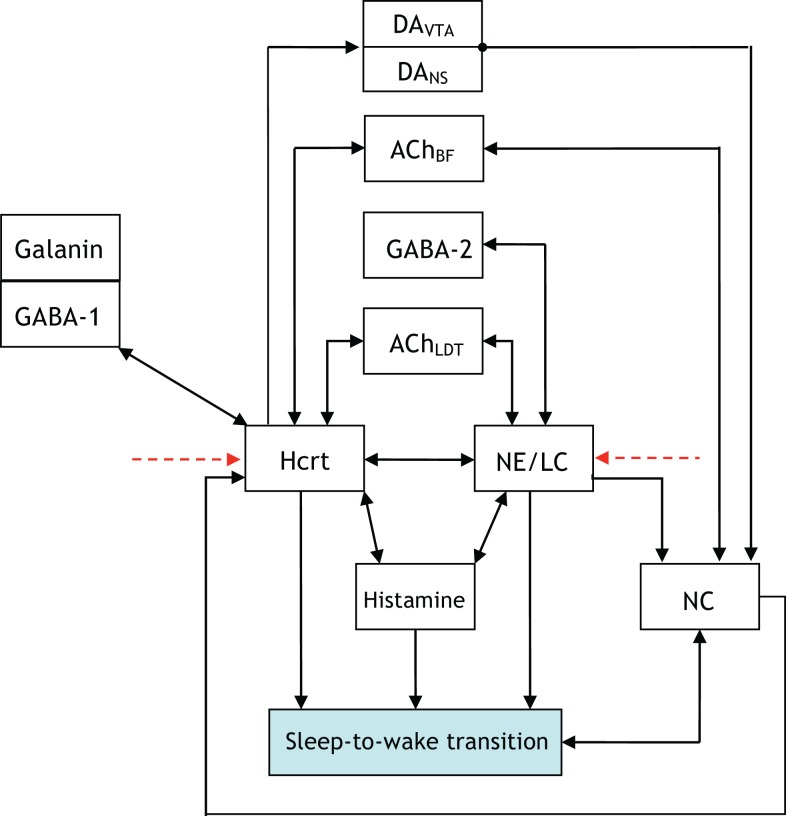
**Updated to show additional interactions between the neural circuits associated with the acetylcholine (ACh) neurotransmitter being released in the basal forebrain (BF) and the laterodorsal tegmental (LDT) nucleus**.

The presentation of Figure 4 stemmed from the necessity of formulating a more unified framework for the interaction between NE/LC and Hcrt, and including a neural circuit for histamine producing neurons. Histaminergic neurons are excited by Hcrt-producing neurons via two different timeframes: fast transmission through glutamate receptors and longer lasting Hcrt signaling through Hcrtr2 receptors (51). Histaminergic cells are known to contribute to the stability of the arousal state (52). Two populations of GABAergic neurons, referred to here as GABA-1 and GABA-2, constitute essentially different populations of local inhibitory neurons: GABA-1 modulates Hcrt output (represented by a pool of MCH, LepRB, and neurotensin neurons) (47, 53, 54) whereas the GABA-2 consists of neurons controlling the output of noradrenergic cells in the LC (55). In fact, a recent paper shows that LepRb^Nt^ neurons in the lateral hypothalamus can hyperpolarize Hcrt neurons through a GABA-independent mechanism possibly mediated by K_ATP_ channels (56). Figure 4 illustrates this latter point about the GABA-producing neurons. Neuromodulation of cholinergic systems is known to correlate with arousal (57) and a recent model has linked ACh release to the synaptic plasticity associated with sleep (58). In Figure 5, ACh_BF_ and ACh_LDT_ denote two populations of ACh neurons (from the BF and mesencephalic), which have different firing properties, neuronal connectivity, and expression of Hcrt receptors (3, 59). Dopamine (DA) has also been implicated as a player in sleep and arousal. The addition of DA producing neurons by the VTA and the nucleus accumbens is shown in Figure 5 to reflect the mesocorticolimbic and nigrostriatal pathways, respectively (60–66).

**Figure 6:**
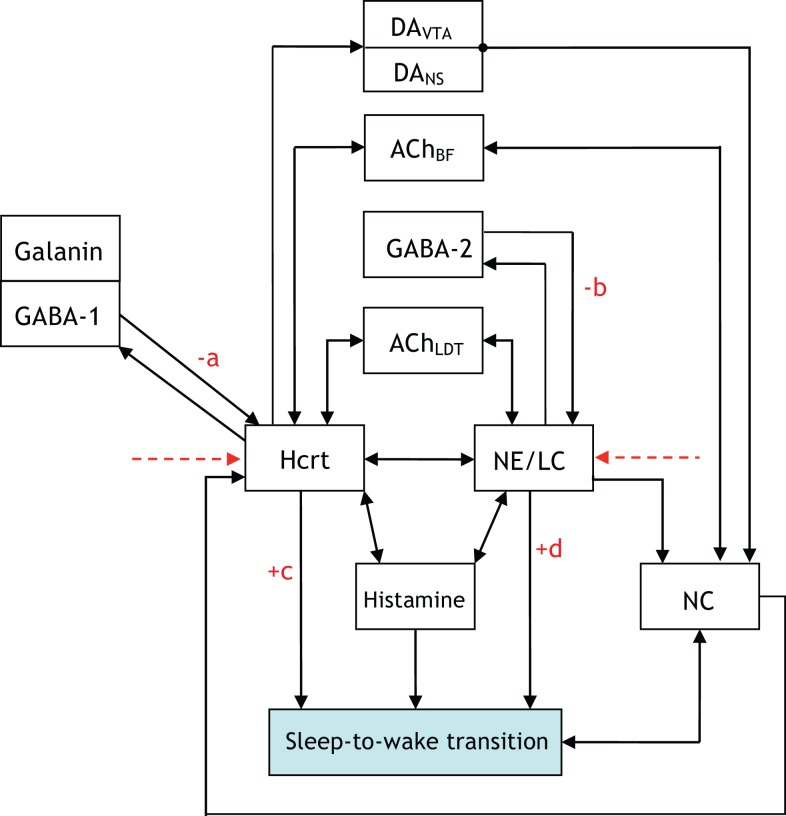
**An updated version of Figure 5, which contains +/− on various connections to denote whether the interaction promotes/impedes the sleep-to-wake transition**. We hope research progresses to a point where the degree of the promoting/impeding is quantified through a scoring system with the pictured constants a, b, c, d assigned scores from 1 to 10.

The five figures presented thus far illustrate neuronal interactions within the context of a functioning biological system. A comprehension of the system would entail iterating, re-formulating, and expanding upon the figures. Aside from adding blocks and augmenting the presented figures; it may be equally appropriate to disregard blocks not relevant to particular experiments. A prospective enhancement to the interactions illustrated via Figures 1–5 would be to label the arrows with a “+” or “−” to indicate if the interaction promotes or impedes a sleep-to-wake transition, respectively. For instance, it is conceived that the GABA-1 to Hcrt arrow and the GABA-2 to NE/LC arrow should have a “−” sign associated with them. A scale of 1–10 can be conjoined with the sign to quantify the degree of the promotion or impediment. Such a scoring system allows for a quantitative differentiation of one neural circuit as potentially exhibiting a level of hierarchy with respect to another circuit. For instance, the NE/LC to sleep-to-wake connection and the Hcrt to sleep-to-wake connection in Figure 6 would both receive a “+” since they promote the transition. However, the former will have a higher score associated with its positive sign because NE/LC has been shown to hierarchically gate Hcrt during the transition. The determination of such scores will evince the strength of the coupling and the inherent hierarchy among the neural circuits. As mentioned in Figure 6, this constitutes a far-reaching goal of research in the sleep community.

**Figure 7:**
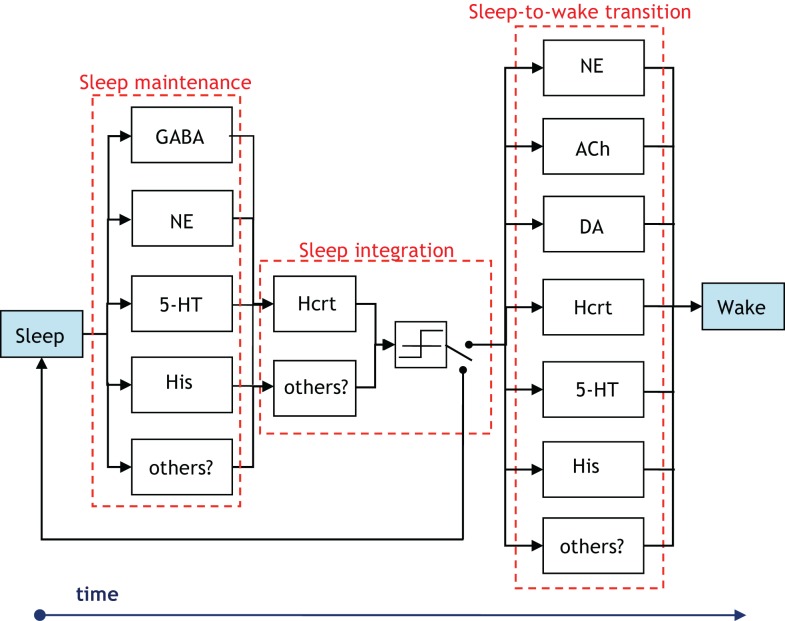
**Illustration of the groups of neurons associated with the temporal progression of the sleep-to-wake cycle**. The presence of vertically parallel blocks indicates an inherent degree of redundancy among the neuronal groups, which are involved in the procurement of that specific stage (i.e., sleep maintenance, sleep integration, or sleep-to-wake transition). The GABA block in the sleep maintenance stage denotes GABA-producing neurons in the neocortex, hypothalamus, and brainstem. Interestingly, the NE and 5-HT producing neurons influence sleep maintenance more so by being silent than by firing. A switch is posited to exist following a threshold device in the sleep integration stage. The output of the threshold device drives the switch to induce either a sleep-to-wake transition or continued maintenance of the sleep state.

There is presently a paucity of data to evince which neuronal systems drive the activity of others in the procurement of sleep and arousal. Thus, despite the definition presented in Figure 3, the notion of hierarchy is used with discretion in the ensuing sections. We unequivocally advocate experimental research that either proves or disproves the notion of certain neural circuits hierarchically gating other neural circuits in the induction of a sleep-to-wake transition. Concurrently, a crucial distinction must be made while we discuss hierarchy between the neural circuits involved in the sleep–wake cycle. Neurotransmitters will have disparate roles depending on whether we consider a sleep-to-wake transition or the maintenance of sleep. Note that a neurotransmitter silent during sleep may have an important role in sleep maintenance if low levels of its activity cause an awakening (32, 67). This may be the case for NE as permissive activity of LC neurons will likely lead to sleep fragmentation. Figure 7 is presented to delineate the neuronal populations associated with a sleep-to-wake cycle. In Figure 7, the parallel blocks at a temporal juncture denote redundancy among the neuronal groups which are involved in the procurement of that particular stage. Although redundancy will be discussed in Section “The Role of Redundancy in Sleep and Wakefulness,” we remark that the presence of only Hcrt-producing neurons during the sleep integration stage indicates that Hcrt is the sole neurotransmitter governing the shift from NREM sleep to the commencement of a sleep-to-wake transition. The Hcrt system will have a predominant role in our discussion as biological evidence supports a critical, non-redundant, integrator function of these neurons in coordinating other neural circuits to achieve stability of vigilance states. In contrast, other operations regarding maintenance and onset of wakefulness are likely conducted through a redundant network of systems, as discussed below.

### The role of feedback in sleep and arousal

From an engineering perspective, feedback between the neural circuits of the prior subsection is crucial for healthy operation of homeostatically driven systems such as sleep/wake cycles, feeding, and thermoregulation. This is due to the important role that feedback plays in control systems and communication networks (68). A large body of analytical work has modeled the utilization of various forms of feedback (e.g., perfect, noisy, lossy, quantized, delayed) for an eclectic array of purposes. Of equal interest are the objectives for which causal feedback signals have been used. In a control system, feedback is used to drive the system to a particular state or to stabilize a potentially unstable system. Conversely, feedback signals are used in communication networks and signal processing algorithms to either improve a criterion or to assimilate information about parameters that will eventually lead to the improvement of a metric. Recent works such as Doyle and Csete (69) have commenced a progressive discussion on the degree of robust control that must be innate to a functioning CNS as an organism behaves. The aforementioned work has highlighted the important point that feedback within a group of neural circuits may be best served as comprising a portion of a robust, rather than an optimal, control system. It is exciting and productive that recent works in the sleep community (70) are heeding such system-engineering views and building upon them by suggesting neural populations as performing computations akin to generators, regulators, and comparators.

**Figure 8:**
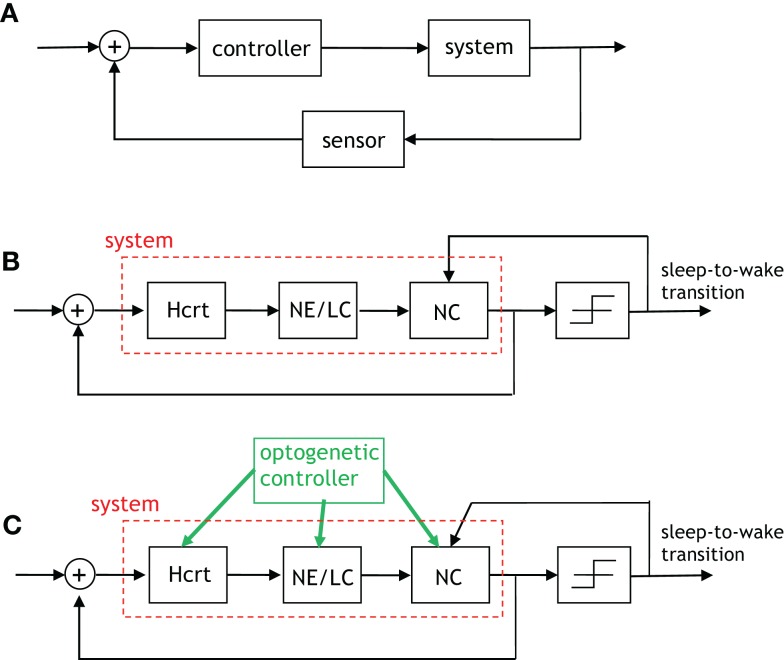
**An algorithmic explanation for the role of feedback in the sleep–wake system**. **(A)** A generic control system with output feedback. **(B)** This figure depicts the feedback provided by the neocortex to Hcrt neurons (referred to as state feedback) as well as the feedback from the occurrence of a sleep-to-wake transition to the neural activity in the neocortex (referred to as output feedback). The right-most block is a threshold device, which outputs a signal contingent on its input exceeding/not-exceeding a specific threshold. **(C)** An “engineered” version of the prior schematic with the green arrows indicating where optogenetic stimulation/inhibition can be applied so as to drive the feedback control system to particular states and induce an ensuing behavior.

We will restrict attention to two feedback loops that are present in Figures 4–6: the feedback signal from sleep-to-wake transition to the NC, and a feedback signal from the NC to Hcrt. It is productive to describe such signaling via incipient models that will be proximately advanced to capture more detailed and elegant aspects of the biological network. The uppermost schematic in Figure 8 depicts a rudimentary feedback control system discussed at length in textbooks such as Brogan (71). The schematic shows what is referred to as output feedback because the output of the system is relayed to the input of the controller for subsequent processing. The purpose of the first schematic is to motivate the middle schematic of Figure 8, which is a control-theoretic model for the two feedback loops that we have mentioned. Interestingly, both output feedback and state feedback are necessary to describe the neuronal interactions taking place even in such a simple depiction of this complex process. The signal from the NC to Hcrt depicts the state feedback, and the sleep/wake signal relayed from the output of the threshold device to the NC depicts the output feedback. It is important to note that Figure 8B does not contain a discernible controller. A controller (or several coupled controllers) must exist for proper neurological operation; however, such controllers are embedded in the system rather than being entities that can be optimized via an engineering procedure. A controller can be instantiated within the neural system by optogenetically applying extrinsic stimulation/inhibition to the neuronal populations as shown in the lower schematic of Figure 8. It should be apparent that this schematic is a gross simplification of the models that we have discussed in Section “Modeling Neural Circuit Interactions During Sleep-to-Wake Transition,” for instance, the direct interaction from the NE/LC to Hcrt neurons has not been considered in Figure 8. Nevertheless, the utility of considering such a scheme is its conduciveness to real-time control of the three neurological units. In fact, preliminary results from various points in this schematic have been studied by recording behavior and EEG signals in Carter et al. (30, 32).

In addition to the intrinsic feedback circuits presented above, other feedback sensors must exist providing information about external factors, including metabolic input and circadian time. These variables may be integrated at many different points within this circuit. For instance, Hcrt neurons are primarily sensitive to dietary amino acids (72), and indirectly sensitive to other metabolites such as fat because of their interaction with LepRB cells (54). Similarly, circadian input may be received from the suprachiasmatic nucleus (SCN), its output relay in the dorsomedial hypothalamus, or alternatively may be sensed by transcription of molecular components of the circadian clock (i.e., *B-mal*, *Per*, etc.) within the integrating neuronal systems(73).

### The role of redundancy in sleep and wakefulness

The notion of redundancy in engineered systems is largely driven by the desire to introduce reliability into systems that are subject to error owing to external perturbations. Various operations are repeated in an organized manner so as to provide a robust design that mitigates the detrimental effects of the stochastic perturbations. Redundancy has been studied for decades within the coding and information theory community [see texts such as Wicker (74) and Gallager (75) and shall be utilized in our discussion]. It will become apparent that a judicious application of the concepts from the coding theory literature to the neurological realm must be practiced as many of the classical concepts will not directly apply.

Redundancy exists in the sleep-regulatory system since it has been shown that the system can function despite the inactivation of constituent neural circuits. A caveat in the analytical research of this redundancy is that it has been designed and implemented by nature. Consequently, we are faced with an inverse problem of identifying the characteristics of redundancy that exists in a functioning system rather than designing the redundancy and analyzing the ensuing performance. In summary, we have encountered the nuance that the system has been formed by nature rather than designed through a documented procedure. The existence of redundancy among the neuronal populations, which promote sleep and arousal has been suggested in (76) with the authors alluding to an arousal system’s compensation to a chronic loss of components. Lesion studies in rodents by Shiromani and colleagues (77, 78) have also motivated the need to consider models for redundancy. In Blanco-Centurion et al. (77) the BF cholinergic, TMN histamine, and LC noradrenergic neurons were collectively lesioned via application of saporin-based neurotoxins, and evidence was provided for the lesioned rodents having decreased transitions between the sleep–wake states indicating a less fragmented and more stable sleep–wake architecture. Due to the majority of this presentation being aimed at sleep-to-wake transition, the modeling herein will not address the long-term stability of the sleep–wake architecture, but unequivocally advocate stability analysis as a future direction. In this subsection we strive to formalize redundancy by presenting a methodology to model such hypothesis. It should be emphasized that our formulation is largely possible because optogenetics permits the functionality of the constituent neural circuits in the sleep-regulatory system to be studied.

**Figure 9:**
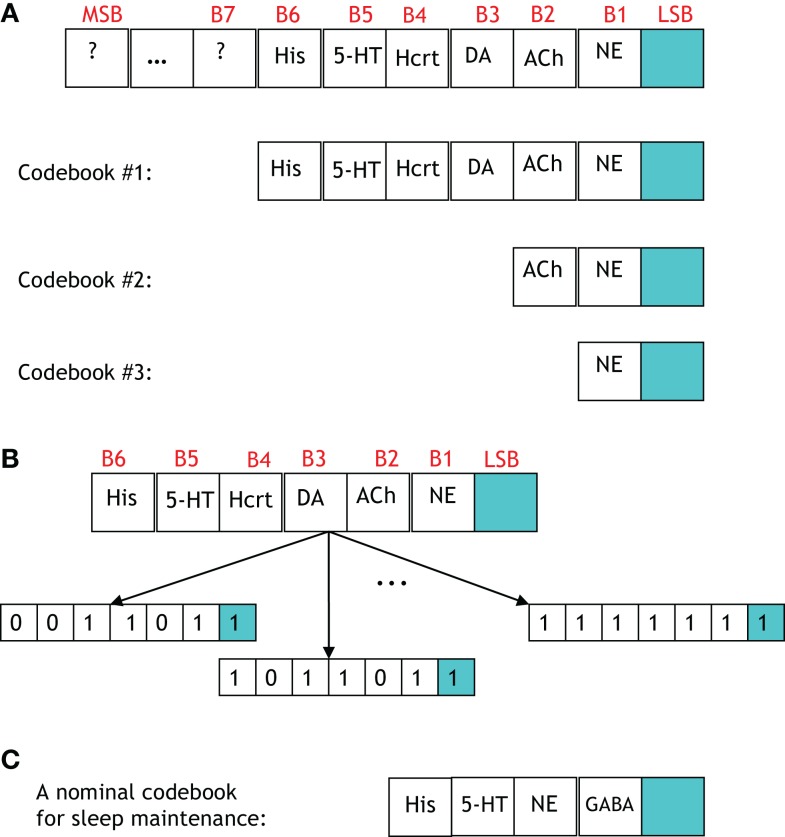
**(A)** A general codebook (top) and three more specific codebooks for modeling the notion of redundancy among the neural circuits governing a sleep-to-wake transition. The following definitions exist for the individual bits in each codeword. LSB: behavioral state (LSB = 1 denotes the wake state, and LSB = 0 denotes the sleep state), B1: NE activity exceeding a threshold, B2: acetylcholine activity exceeding a threshold, B3: dopamine activity exceeding a threshold, B4: Hcrt activity exceeding a threshold, B5: serotonergic activity exceeding a threshold, B6: histamine activity exceeding a threshold. **(B)** Three codewords from Codebook #1 are shown. The leftmost codeword represents the scenario where the NE, DA, and Hcrt activity levels exceed an activity threshold; but the ACh, serotonin, and histamine activity do not exceed such a threshold. **(C)** A specific codebook for representing the redundancy among the neural circuits involved in sleep maintenance is suggested.

A study of the number of neural circuits necessary to induce a sleep/wake state with a particular probability begets the discussion of a codebook. Figure 9 portrays four codebooks, which enumerate the components involved in inducing arousal. The 7-bit codebook labeled Codebook #1 contains 2^7^ = 128 codewords. As shown in Figure 9A, the least significant bit (LSB) denotes the procured behavioral state. The bits that precede the LSB are arranged in a decreasing order of importance with respect to the respective neurotransmitter inducing a sleep-to-wake transition. For instance, the fact that LC (and its synthesis of NE) is perceived to hierarchically gate Hcrt in the sleep-to-wake cycle leads to the former being assigned bit position B1 and the latter B4. ACh has recently been shown to be a powerful initiator of sleep-to-wake transitions (79), although optogenetic stimulation of the BF seems to be less effective than NE/LC at promoting wakefulness. This has been reflected in the framework of Figure 9A with the corresponding bit assignments of B2. Similarly, works such as Isaac and Berridge (80) and Wisor et al. (81) have demonstrated a prominent role of DA in sleep/wake transitions, also Lazarus et al. (64) have justified DA in the basal ganglia as being crucial for sleep-to-wake regulation thus promoting the assignment of DA to B3.

We define the probability that the wake state (i.e., LSB = 1) occurs with absolute certainly by P[LSB = 1] = 1, and define the probability of the *i*th codeword inducing a state on a subject by P[Ci]. The degree of redundancy in the system is conveniently quantified through our codebook paradigm since the occurrence of the sleep/wake state can be expressed as a single probability with a minimal representation of 2 bits^1^. An increase in the reliability of inducing sleep/wake is attained by using 3 bits per codeword or, in other words, increasing the redundancy. The cases of 2 and 3 bits per codeword correspond to the respective consideration of Codebook #3 and Codebook #2 in Figure 9A. An example of three codes from Codebook #1 is shown in Figure 9B, and the neurological ramifications of the three codes are quantified by the following conditional probabilities:

$$\begin{array}{llll} & P\left[\mathrm{LSB}=1|\mathrm{NE}=1,\mathrm{ACh}=0,\mathrm{DA}=1,\mathrm{Hcrt}=1,\mathrm{5-HT}=0,\mathrm{His}=0\right],\; & & \;\\ & P\left[\mathrm{LSB}=1|\mathrm{NE}=1,\mathrm{ACh}=0,\mathrm{DA}=1,\mathrm{Hcrt}=1,\mathrm{5-HT}=0,\mathrm{His}=1\right],\; & & \;\\ & P\left[\mathrm{LSB}=1|\mathrm{NE}=1,\mathrm{ACh}=1,\mathrm{DA}=1,\mathrm{Hcrt}=1,\mathrm{5-HT}=1,\mathrm{His}=1\right].\; & & \; \end{array}$$

The events, which are conditioned upon above denote biological states that have been optogeneticaly induced via stimulation/inhibition. Determination of the 2^7^ = 128 conditional probabilities accompanying the codewords in Codebook #1 would elucidate the coupling among the neuronal populations and the sleep-to-wake transition depicted in the block diagrams of Figures 4–6.

There are important points to be wary of when investigating the neuronal redundancy in the sleep and arousal system with the aid of coding theory. For instance, from a coding-theoretic prospective, the codebooks that we have presented provide no redundancy. This is because, taking Codebook #1 as an example, the 2^7^ = 128 codewords deployed by the system is not less than the number of possible 7-bit sequences (which is also 2^7^ for a binary alphabet). Furthermore, the so-called minimum distance of Codebook #1 is equal to one which portends a lousy codebook if the objective was to ultimately perform a decoding operation on a codeword transmitted along a noisy channel. However, that is not the goal here, and the reliability provided by the codeword is not measured by decoding accuracy but rather by adeptly inducing a behavioral state^2^. Alternatively, we define the redundancy of Codebook #1 in terms of the number of units incorporated in the induction of a behavioral state. With such an alternate definition Codebook #1 provides redundancy of degree seven because six (binary-valued) units are utilized to procure a single (binary-valued) behavioral state.

To progress the above model we must consider several examples where specific codewords from Codebook #1 are compared. We begin by comparing the two codewords:


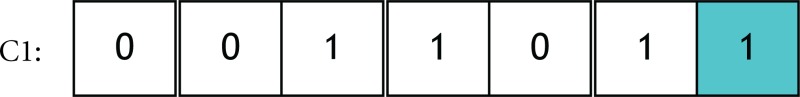



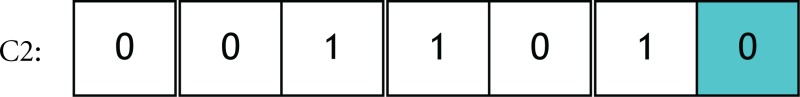


If P[C1] > P[C2] then it will be stated that C1 probabilistically dominates C2. Neurologically, this indicates that when the activity of NE, DA, and Hcrt neurons concurrently exceed specific (and predefined) thresholds, a wake state will occur with higher likelihood than a sleep state. We assert this as a sensible deduction since NE/LC hierarchically gates ACh in eliciting sleep-to-wake transitions. If a comparison is made between two codewords that induce the same behavioral state such as when comparing:


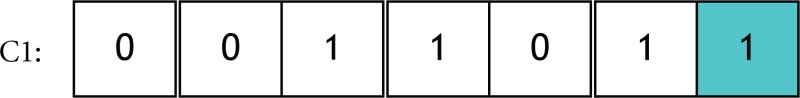



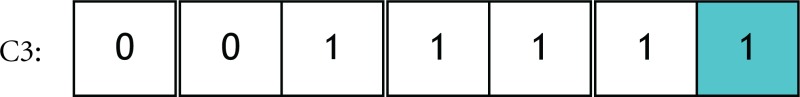


then it is expected that C1 will probabilistically dominate C3 (that is, P[C1] > P[C3]) because an additional activation of ACh neurons has been shown to promote REM sleep. A more provocative finding would be expected when comparing the following two codewords:


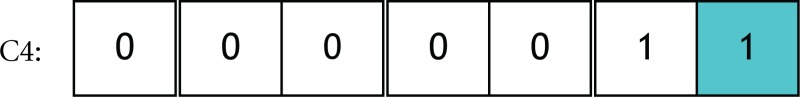



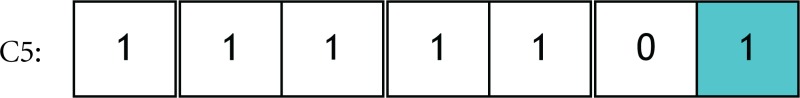


where it is not intuitively apparent, which codeword would be dominant. In summary, we have proposed an analytical methodology by which the interactions of the neuronal populations can be quantitatively compared in their induction of a behavioral state. We remark that, in general, it is not sensible to perform the above comparisons among two codewords, which have different non-LSB bits and a different LSB bit (e.g., C2 and C3). This is because it is rarely possible to make definitive statements about the modulatory effects of neural populations when comparing scenarios that concurrently portray different neuronal activation patterns and different induced behavioral states.

The framework of a codebook with codewords probabilistically dominating each other in the procurement of a behavioral state can provide neuroscientists the capability to identify what coding-theorist refer to as algorithmic “structure” among the neural circuits, which cause the sleep or arousal state. More precisely, if there is a discernible pattern or relationship among groups of codewords, then it will be stated that the codebook exhibits structure. For example, if codeword C4 were to probabilistically dominate C5 then it would be expected that C4 would also probabilistically dominate all 2^5^ = 32 codewords of the form:


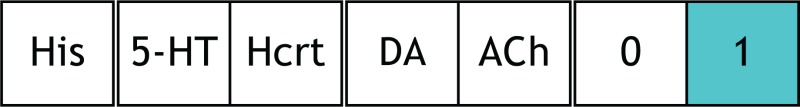


where the constituent ACh, DA, His, Hcrt, and 5-HT populations could be either activated (i.e., assume a value of “1” in the codeword) or quiescent (assume a value of “0”) without affecting the LSB value. The above example is a simple instance of identifying inherent structure within a codebook. Discovery of structure in a codebook would certainly be valuable in providing insight into the collective operation of the components in Figures 4–6. We shall discuss and further motivate optogenetic experiments that are directed at discovering such structure in Section “Suggested Experiments.”

Another biologically elucidating avenue for investigation is determining the amount of redundancy present in the sleep regulating system. We shall describe how such a study can be undertaken with the aid of the above framework. Our example will encompass two codebooks that are simple extensions of each other. Consider Codebook #2 in Figure 9A with the following probabilities for the induction of the wake state:


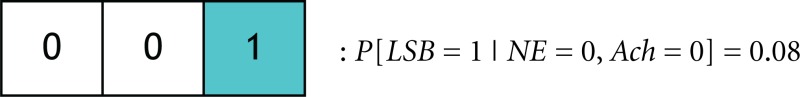



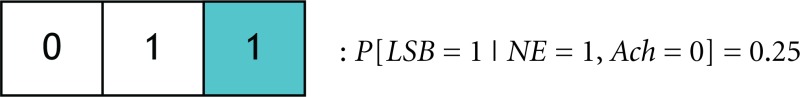



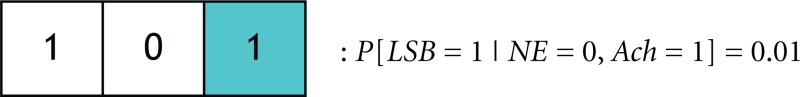



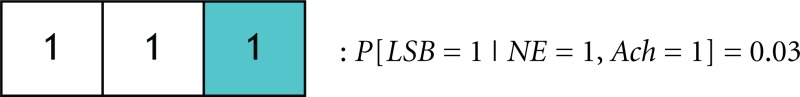


If we now consider a four-bit codebook that is an appended version of the three bit codebook above (in this case, DA will be the additional bit appended as B3), then the codewords that induce the wake state are given by:


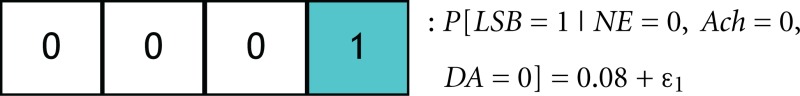



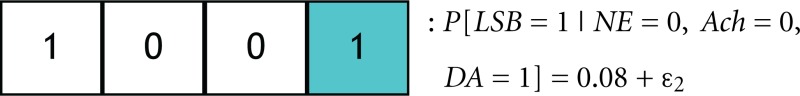



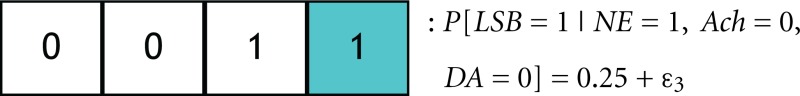



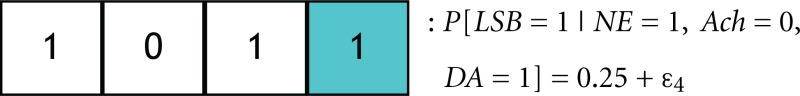



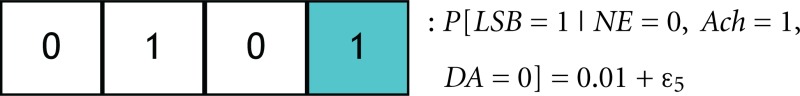



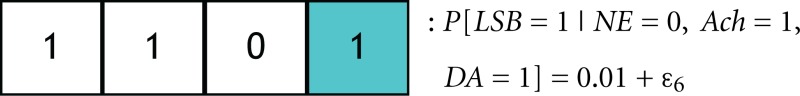



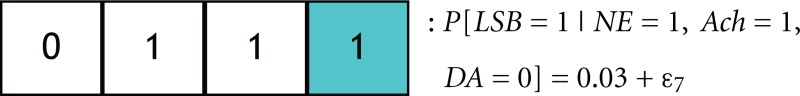



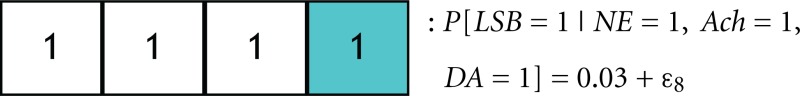


The quantities ε_1_, ε_2_, ε_3_, ε_4_, ε_5_, ε_6_, ε_7_, and ε_8_ represent positive or negative adjustments to the probabilities of the (previously listed) three bit codewords in order to yield the wake probabilities for four-bit codewords. In the case that the adjustments are sufficiently small^3^ it may be deemed that the addition of the fourth bit, in this example the consideration of the DA releasing neurons, is an inconsequential component to the induction of the wake state. In such a scenario, it would be sensible to posit that a three bit codebook contains the sufficient amount of redundancy that the sleep-regulatory system has been “programmed” to use.

Interestingly, the efficiency of a network is a notion, which is frequently at-odds with redundancy. In other words, it may be argued that the sleep-regulatory system is efficient in its capability to prospectively compress a 7-bit codebook into a 2-bit codebook while sacrificing a negligible amount of reliability in inducing a behavioral state. Also, it should be noted that a reduction in redundancy (i.e., compression) will not annihilate the biological operation, but rather impair its nominal reliability in probabilistic fashion. The interplay and trade-off between redundancy and efficiency within the context of the neural circuits governing sleep-to-wake transition is an avenue which promises to yield luminous interpretations when studied in the future. Similarly, a number of codewords that do not appear to be essential initially may need to be added for stability. The discussion of this section has been within the context of the induction of a sleep or wake state. We believe the theme of redundancy should also be addressed within the context of sleep maintenance. In Figure 9C we have suggested a preliminary codebook for depicting the relative importance of various neurotransmitters in sleep maintenance.

## Suggested Experiments

Affirmation of the ideas presented in this work inevitably requires experimental investigation. By virtue of the drive theme discussed in Section “Introduction,” we shift attention to providing guidance on experiments that provide insight into the construction and function of the sleep-regulatory system. Although we take a high-level approach, the prospective experiments that will be discussed here are intended to instantiate the theory considered above. The suggested experiments draw heavily upon what we shall refer to as combinatorial optogenetic studies. This includes the selective stimulation/inhibition of different combinations of neural circuits in order to induce a sleep-to-wake transition.

The primordial set of experiments that we suggest would entail discovering whether the neural circuits depicted in Figure 6 interact to induce or impede the sleep-to-wake transition. Although we suggest what we have spawned in Section “Modeling Neural Circuit Interactions During Sleep-to-Wake Transition” as a primordial item to be determined via experimentation, the resolution of a +/− for the connections in figure may not be categorical. This is because neurotransmitters such as histamine will not have an exclusively inhibitory or excitatory effect on the efferent neural circuits. Conduction of experiments that quantify the degree of the induction/impeding via the scoring system discussed in Section “Modeling Neural Circuit Interactions During Sleep-to-Wake Transition” would be a sensible ensuing item to consider.

The experiments performed in Carter et al. (32) have begun to address the notion of a neural circuit hierarchically gating another in the induction of a sleep-to-wake transition. To test for hierarchical gating the definition illustrated in Figure 3 would need to be applied on a pair-wise basis to the neural circuits portrayed in Figure 6. Intelligent selection of sequences of pair-wise combinations to stimulate/inhibit may elucidate the existence of the hierarchy among the neural populations, which are engaged in a sleep-to-wake transition. Although a concrete definition for hierarchical gating has been presented in Section “Modeling Neural Circuit Interactions During Sleep-to-Wake Transition” and experiments for determining the hierarchy have been discussed; we remark that the general idea is still a hypothesis. Hence, proof-of-existence experiments vindicating the hierarchy hypothesis would serve as scientific contributions to the sleep community. In addition to the pair-wise comparisons being investigated via optogenetic experiments, one could also explore the inactivation of genes associated with a particular neurotransmitter. An example of this strategy is reported in Carter et al. (82) where optogenetic stimulation of Hcrt cells led to c-fos stimulation of several brain regions including NE/LC and Histaminergic TMN (but not serotoninergic cells in the raphe). Interestingly, in spite of evidence showing that Hcrt provides excitatory input to histamine neurons (51), the absence of histamine in HDC knockout mice did not prevent Hcrt-induced awakenings strongly suggesting that histamine does not hierarchically gate Hcrt.

The notion of feedback discussed in Section “The Role of Feedback in Sleep and Arousal” may also be analyzed experimentally using iterative combinations of optogenetic stimulations and inhibitions. Figure 8C depicts the convergence of signals in the NC as providing the necessary incitement for a behavioral state transition. It is known that neocortical activity during sleep is characterized by delta waves in the EEG, which purportedly indicate synchronous firing of pyramidal neurons. Intracellular recordings by Steriade and colleagues during sleep indicate the presence of up-and-down states of membrane depolarization and irregular firing patterns of pyramidal cells (83). Recent data by Tononi and colleagues (84) suggest local events of sleep-like states during wakefulness, which become more prevalent as the animal approaches full sleep. A testable hypothesis suggests that subcortical arousal networks, which include the elements depicted in Figure 8, would be essential to providing coherence to the cortical domains that experience local brain states. Thus, a worthwhile initial experiment would entail the application of a short pulse to stimulate the NE producing neurons in the LC and confirm that the activity is sufficient for eliciting changes across the entire neocortical volume in a short time. Conditional mouse mutants with disrupted neuronal activity in local areas of the cortex may also provide a valuable tool for testing these hypotheses. The aforementioned constitutes the model in Figure 8 driving a series of experiments aimed at elucidating a biological process.

An equally elucidating series of experiments for providing insight into the role of feedback in Figure 8 would entail the combinatorial study of optogenetically applying the eight possible stimulation/inhibition patterns to the three neural circuits manipulated by the optogenetic controller. The following are a few of the questions that would be answered by such a study. Does a particular stimulation/inhibition combination lead to faster operation of the biological feedback system in Figure 8? Does a specific stimulation/inhibition combination cause oscillatory behavior? Will one of the neural circuits prevail by dictating the operation of this feedback system?

The prospective redundancy among the interacting populations of neurons that coordinate sleep-to-wake transition begets an interesting series of proposed experiments. Optogenetics and chemogenetics provide the cell-selectivity and temporal resolution necessary to study complex interaction between neural circuits. In Section “The Role of Redundancy in Sleep and Wakefulness,” we have provided rather simple examples of two sets of experiments, which will bestow insight to the sleep community. These are (1) experiments to identify the structure among the neural circuits which cause the sleep or arousal state, and (2) experiments which determine the amount of redundancy present in the sleep regulating system. The first set of experiments cannot be undertaken without the guidance of the codebook model’s capability to represent relationships among optogenetically induced ordered sequences. The framework will allow a means of studying prospective patterns (which are expected to exist) by methodologically stimulating/inhibiting sequences of neural circuits. A fascinating aspect of the second series of experiments is the insight that the anticipated discoveries will yield on the functional structure extant in the sleep-to-wake circuitry. Each codeword in Figure 9 is attained by selectively stimulating/inhibiting a precise set of the neural circuits discussed in this paper. Performing combinatorial optogenetic experiments with the intent of determining, which components of the codebook do not have a probabilistically significant role in a transition will determine the redundancy present in the sleep-regulatory system. This is an incipient step in answering the question of whether nature chose to favor redundancy or efficiency in its creation of this system. The presence of a few crucial elements (e.g., NE, ACh, DA producing neurons) in the codebook would favor efficiency; while the presence of a larger number of elements would favor redundancy. We certainly expect the answer to this question to be debatable upon completion of the proposed combinatorial optogenetic experiment. Nevertheless, empirical knowledge into the opposing sides of this argument will arise and be vetted through the codebook model.

A necessary item must accommodate the experiments, which investigate redundancy. The thresholds for the codeword components to be assigned values of “1” would need to be disclosed. Since a binary alphabet is assumed for the components of the codewords, an N-bit codebook would require specification of N-threshold values. The physical interpretation of the thresholds is that they represent the firing frequency necessary to declare a neural population as having been stimulated. It is natural to define such firing frequency as the collective spike rates of all the neurons in the neural circuit in question. To further complicate matters, the threshold values associated with the neural circuits would contain some degree of variability across different species and even genetically altered versions of the same species. Nevertheless, this is a requisite for amassing a codebook.

Although a specific hierarchical construction is promoted in the codebook formulation and Figure 9; we stress that the ordering of the elements is still primitive and uncertain. We expect forthcoming experimental studies to encourage different orderings of the elements in the codebooks. A benefit of the presented model is the malleability of the codebooks. This is highly advantageous in allowing experimenters to propose adjustments as more data becomes available. The ordering of the elements in the codebook would be determined by the hierarchy study discussed above, and as increasing evidence for various hierarchical relationships are disclosed, the elements, which comprise the codebook will be appropriately rearranged.

## Conclusion and Future Work

Sleep-to-wake transitions are regulated by a variety of subcortical neuromodulatory systems. It is accepted that the interaction and coupling of the involved units are rather complex. The authors (29, 67, 82, 85) have provided results to instantiate the coalescence of optogenetics and judicious analytical modeling. Prior works have supplied insight into the contribution of various neuromodulators in the dynamics of brain state transition. In this paper, we have shifted focus to present a framework that has a potential for unifying future experimental and modeling efforts aimed at understanding the dynamics of the sleep-regulatory system. We have added additional components to the computational model that was originally presented as part of Carter et al. (32) in order to better understand the role of GABAergic and histaminergic neurons. The voltage traces in Figure 2 exhibit that GABAergic neurons improve the coherence of Hcrt and NE/LC neurons by abetting synchronization among those two populations. A more interesting scenario is illustrated in the voltage traces of Figure 4 where GABAergic neurons intervene to maintain system stability by controlling the depolarizing effect of histamine. This is an appeasing finding in light of the idea that the mechanisms promoting gain control in neural systems require a tight balanced between the depolarizing and hyperpolarizing forces, which operate on multiple brain circuits (44–46).

The role of glia in sleep is far from being comprehensively understood. Through gliotransmission astrocytes modulate the accumulation of sleep pressure and hence influence slow-wave activity (SWA). More specifically, the ATP released by astrocytes is rapidly hydrolyzed to form adenosine, which has been shown to increase sleep and enhance SWA (86, 87). Furthermore, the neurotransmitter-based dialog between neurons and glia has been mentioned as modulating neuronal excitability (88). Works such as Amzica et al. (89) have provided evidence for glial cells spatial buffering extracellular K^+^ during normal slow sleep oscillations. It is apparent that the analytical modeling of sleep-to-wake transition and sleep maintenance would be incomplete without the model definitively including the functionalities of glia and consequences of glia-neuron interactions.

The modeling of sleep coherence, as defined by synchronization of cortical modules or local domains, is more nascent and perhaps more elusive than the sleep-to-wake transition and sleep maintenance realms that we have discussed. Many questions arise from local sleep phenomena, such as the existence of a threshold for overall cortical synchronization, and whether this global sleep oscillation is necessary for the biological functions served by sleep. The extension of the redundancy, hierarchy, and feedback concepts to sleep coherence is beyond the scope of the present work, but certainly an important and promising future avenue.

The neural populations discussed in this work contribute different computational operations to the disposition of being awake or sleep. We have presented a framework to model the neurotransmitter interactions in a sleep-to-wake transition. The framework commenced with architectures identified in order of increasing complexity as each representation subsumed additional populations of neurons. Subsequently, we proposed definitive models to accompany the notions of hierarchy, redundancy and feedback in the sleep-regulatory system. Even though the structure of the redundancy and the prospective hierarchy of the complex network discussed here is largely undiscovered; a constructive step has been taken by introducing a framework to model such themes as further data becomes available. We have stressed that our analytical framework is motivated by advancements in optogenetics, which allow for the manipulation of the transition. Reciprocally, it is expected that the modeling considered herein will be conducive in interpreting, assisting, and driving the subsequent research that uses optogenetics to study the sleep-arousal system. We hope to have motivated researchers to draw upon our algorithmic concepts and perform experiments to provide data, which would augment or perhaps revise our ideas. We also invite corresponding works to present advancements to the presented models or alternative models.

While providing a review of existing models is not the objective of this work; it is insightful to discuss two extant modeling efforts (in addition to the already discussed flip-flop model of Saper and colleagues) and how they pertain to our formulation. First, the generation of thalamic spindle oscillations and their effects on sleep and arousal was investigated in Refs. (27, 83, 90, 91). Our formulation pertains more to the hypothalamic and brainstem control of cortical systems rather than the thalamic architecture. In fact, it was noted in Ref. (27) that the diversity of the interactions brought on by neurons of the cortical circuitry leads it to be more difficult to model than thalamic networks. Also, the present discussion has not been geared toward the operation of the thalamocortical loops; rather the causal relationships between the interacting neuronal populations that are involved in the wake sleep–wake cycle. Second, the synaptic homeostasis hypothesis described in Tononi and Cirelli (92) has been justified from the physiological perspective of EEG SWA showing an increase during vigilance and a decrease during sleep. More recent works such as (93) have derived large-scale network models to reproduce the dynamics of the synaptic strengthening/depression among the cortical and thalamic networks. While it would be inaccurate to claim orthogonality between the sleep homeostasis hypothesis and the framework presented herein, we remark that the presentations are quite disparate in discussing different aspects of sleep. The analysis herein is tailored toward the effect of optogenetics on populations of neurons, and does not speculate on the synaptic strengths within the neural circuits. Works such as (40, 94) have addressed the integration of sleep homeostasis and Hodgkin–Huxley models for Hcrt neurons. The unification of a large-scale corticothalamic model, which merges sleep homeostasis and synaptic scaling with our notions of hierarchy, feedback, and redundancy, is beyond the scope of the current paper but an exciting future avenue.

## Author Contributions

This work was conceived by extensive discussions between all authors. RH developed the conductance-based modeling and all simulations of voltage traces shown in Figures 1, 2, and 4. SS wrote the manuscript with feedback from RH and LdL.

## Conflict of Interest Statement

The authors declare that the research was conducted in the absence of any commercial or financial relationships that could be construed as a potential conflict of interest.

## Footnotes

^1^The LSB is included in this tally, thus 2 bits is the smallest code-size that can be considered.

^2^Also, there is no discernible noisy channel in this context.

^3^In practice, application of a Neyman–Pearson test would be a sensible means of determining if this is indeed the case.
